# TLR4 Activation Promotes the Progression of Experimental Autoimmune Myocarditis to Dilated Cardiomyopathy by Inducing Mitochondrial Dynamic Imbalance

**DOI:** 10.1155/2018/3181278

**Published:** 2018-06-26

**Authors:** Bangwei Wu, Jian Li, Huanchun Ni, Xinyu Zhuang, Zhiyong Qi, Qiying Chen, Zhichao Wen, Haiming Shi, Xinping Luo, Bo Jin

**Affiliations:** ^1^Department of Cardiology, Huashan Hospital, Fudan University, Shanghai, China; ^2^Department of Cardiology, Zhongshan Hospital, Fudan University, Shanghai, China

## Abstract

Mitochondrial dynamic imbalance associates with several cardiovascular diseases. However, the role of mitochondrial dynamics in TLR4 activation-mediated dilated cardiomyopathy (DCM) progress remains unknown. A model of experimental autoimmune myocarditis (EAM) was established in BALB/c mice on which TLR4 activation by LPS-EB or TLR4 inhibition by LPS-RS was performed to induce chronic inflammation for 5 weeks. TLR4 activation promoted the transition of EAM to DCM as demonstrated by increased cardiomyocyte apoptosis, myocardial fibrosis, ventricular dilatation, and declined heart function. TLR4 inhibition mitigated the above DCM changes. Transmission electron microscope study showed that mitochondria became fragmented, also with damaged crista in ultrastructure in EAM mice. TLR4 activation aggravated the above mitochondrial aberration, and TLR4 inhibition alleviated it. The mitochondrial dynamic imbalance and damage in DCM development were mainly associated with OPA1 downregulation, which may be caused by elevated TNF-*α* level and ROS stress after TLR4 activation. Furthermore, OMA1/YME1L abnormal degradation was involved in the OPA1 dysfunction, and intervening OMA1/YME1L in H9C2 significantly alleviated mitochondrial fission, ultrastructure damage, and cell apoptosis induced by TNF-*α* and ROS. These data indicate that TLR4 activation resulted in OPA1 dysfunction, promoting mitochondrial dynamic imbalance and damage, which may involve in the progress of EAM to DCM.

## 1. Introduction

Dilated cardiomyopathy (DCM) is the most common cardiomyopathy worldwide characterized by ventricular cavity expansion and contraction function declination, which are associated with sudden cardiac death and heart failure [1]. Myocarditis is characterized by pathological autoimmune response of the myocardium, even when asymptomatic, about 10–20% of patients will develop chronic disease eventually leading to DCM [2]. Therefore, an in-depth study into the molecular mechanism underlying the development of myocarditis to DCM is vitally needed for therapeutic interventions of DCM.

TLR4 specifically recognizes and responds to LPS of gram-negative bacteria and participates in multiple autoimmune diseases by acting as a bridge between natural immunity and acquired immunity [3]. Numerous studies have demonstrated that TLR4 activation-mediated leucocyte infiltration in myocardium leads to cytokine secretion, oxidative stress, and protease release, which play a pivotal role in myocardial infarction, ischemia-reperfusion injury, myocarditis, and heart failure [4, 5]. TLR4 and downstream-signaling activation showed importance for the induction of autoimmune myocarditis model [4, 6]. TLR4-deficient mice had significantly reduced myocarditis severity, viral replication, and IL-1*β*/IL-18 after infection with enteroviruses such as CVB3 [7]. Activation of TLR4 on dendritic cell (DC) is required for the induction of acute myocarditis and heart failure [2, 8]. Besides, Satoh et al. showed that myocardial expression of TLR4 was associated with enterovirus replication and LV dysfunction in human DCM [9]. Furthermore, patients with DCM carrying the TLR4 variants rs4986790 and rs4986791 displayed impaired cardiac recovery independently of medical treatment [10]. These results suggest that TLR4 activation could affect the pathophysiology process of DCM, but the underlying mechanism remains unclear.

Mitochondria are central to multiple pathophysiological procedures in energy production, aging, innate immunity, and cell apoptosis [11]. It has been a new focus of researches that mitochondria are highly dynamic organelles and undergo dynamic balance of mitochondrial fusion and fission to maintain normal mitochondrial morphology and function [12]. Mitochondrial dynamics controls metabolic plasticity, redox signaling, and cell death or survival and closely relates with mitochondrial quality control [13]. Mitochondrial fission occurs upon the recruitment of dynamin-related protein 1 (DRP1) on the outer mitochondrial membrane (OMM). Mitochondrial fusion is regulated by dynamin-related GTPases mitofusin-1 (MFN1) and mitofusin-2 (MFN2) isoforms in the OMM and by optic atrophy 1 (OPA1) in the inner mitochondrial membrane (IMM) [12]. The disruption of mitochondrial dynamics is linked to multifarious cardiovascular diseases, such as cardiac ischemia/reperfusion injury, diabetic cardiomyopathy, pulmonary hypertension, and heart failure [14–17]. Moreover, study also indicates that excessive mitochondrial fission in viral myocarditis is related with myocardial injury and apoptosis of cardiomyocytes [18]. However, the role of mitochondrial dynamic balance in DCM development originating from autoimmune myocarditis has not been investigated.

In the current study, we hypothesize that mitochondrial dynamic balance is disturbed in DCM and involved with TLR4 activation-mediated progression of experimental autoimmune myocarditis (EAM) to DCM. We detected cardiomyocyte apoptosis, ROS stress, myocardial fibrosis, and ventricular expansion and function after TLR4 intervention in EAM mice and observed the mitochondrial morphology changes and mitochondrial dynamic-related protein expression. Finally, we explored the possible molecular mechanisms about mitochondrial dynamic change in DCM.

## 2. Materials and Methods

### 2.1. Experimental Autoimmune Myocarditis and Experimental Design

Male BALB/c mice aged 6 weeks were purchased from Animal Experimental Center of Fudan University. The mice were treated with a standard diet and water ad libitum at the Shanghai Medical College Animal Care Facility according to institutional guidelines. For EAM induction, mice were immunized with 200 *μ*L of a 1 : 1 emulsion of PBS with 2 mg/mL of porcine cardiac myosin (Sigma-Aldrich, St. Louis, MO, USA) in complete Freund's adjuvant (CFA) (Sigma-Aldrich, St. Louis, MO, USA) in the groin subcutaneously at days 0 and 7. Sham mice received PBS/CFA only. The EAM mice at fourth week were randomly divided into the following groups: EAM + PBS (intraperitoneal injection of PBS), EAM + LPS-EB (LPS-EB, an ultrapure forms of LPS, was administered intraperitoneally twice a week at a dose of 2.5 mg/kg (InvivoGen, San Diego, CA, USA)), and EAM + LPS-RS (LPS-RS was administered intraperitoneally twice a week at a dose of 2.5 mg/kg (InvivoGen, San Diego, CA, USA)), above intervention lasted for 5 weeks. The study included four experimental groups: sham mice (intraperitoneal injection of PBS only, sham group), EAM + PBS (control group), EAM + LPS-EB (TLR4 activation, LPS-EB group), and EAM + LPS-RS (TLR4 inhibition, LPS-RS group). Sham group mice just received PBS/CFA, and the myocardial inflammatory changes did not occur; control group mice presented myocardial inflammatory changes of EAM and keep a continuous chronic inflammation response. All procedures were performed according to National Institutes of Health Guide for the Care and Use of Laboratory Animals.

### 2.2. Echocardiographic Analysis of Cardiac Function

A VisualSonics Vevo 770 ultrasound machine equipped with a 30 MHz phase array linear transducer was used (VisualSonics Inc., Toronto, Ontario, Canada). The mice were anaesthetized with 2% isoflurane. M-mode images were used to measure LV fractional shortening (FS, %), LV ejection fraction (EF, %), LV end-diastolic dimension (LVEDD, mm), and LV end-diastolic volume (LVEDV, *μ*L), which were acquired by a technician who was blind to the treatment groups.

### 2.3. HE and Masson Staining

Myocardial tissues were fixed in 4% paraformaldehyde, embedded in paraffin, and cut into 6 *μ*m thick slices. A part of the sections was stained with HE, and the other part was stained with Masson. Myocarditis severity was scored on HE-stained sections using grades as described previously [19]. The fibrosis area presents blue; the blue area/total area × 100% indicates the degree of myocardial fibrosis. For quantitation of fibrosis areas, five fields per mouse were analyzed using Image-Pro Plus 6.0 software.

### 2.4. Immunohistochemistry Analysis

The heart tissues were fixed with 4% paraformaldehyde, embedded in paraffin, and sectioned into 6 *μ*m thick slices. To evaluate the expression of OMA1 and YME1L in myocardium, sections after antigen retrieval were incubated with OMA1 antibodies (Santa Cruz Biotechnology, USA) and YME1L antibodies (Thermo Fisher, USA), followed by treatment with horseradish peroxidase-conjugated secondary antibodies. The reaction products were visualized using diaminobenzidine chromogen and counterstained with hematoxylin.

### 2.5. Evaluation of Apoptosis in Tissue Sections and Cardiomyocytes

DNA fragmentation was detected in situ with the use of terminal deoxynucleotidyl transferase dUTP nick-end labelling (TUNEL), as described previously [20]. The hearts were fixed in 4% paraformaldehyde, embedded in paraffin, cut into 6 *μ*m thick sections, and treated as instructed in the In Situ Cell Death Detection kit (Roche Diagnostics GmbH, Mannheim, Germany). The nuclear density was determined via manual counting of the 4′,6-diamidino-2-phenylindole (DAPI)-stained nuclei in five fields for each slice with a ×40 objective, and the number of TUNEL-positive nuclei was determined by examining the entire section with the same power objective, which was performed by a technician who was blind to the treatment groups.

### 2.6. Oxidative Stress Detection

For the detection of oxidative stress in myocardial tissue, DHE staining was performed as described previously [20]. After being harvested, the heart tissues were embedded immediately in an optimum cutting temperature (OCT) compound and stored at −80°C. The unfixed frozen samples were cut into 6 *μ*m thick sections and placed on glass slides. 10 *μ*mol/L DHE (dissolved in DMSO) (Sigma-Aldrich, St. Louis, MO, USA) was applied to each tissue section then incubated in a light-protected humidified chamber at 37°C for 30 min. For detecting mitochondrial superoxide production, H9C2 was cultured with 20 ng/mL TNF-*α* for 12 h after pretreatment with 10 nM MitoTEMPO (dissolved in DMSO) (Enzo Life Sciences, Farmingdale, NY) for 1 h then incubated with 2.5 *μ*M MitoSOX™ Red mitochondrial superoxide indicator (dissolved in DMSO) (Molecular Probes) for 10 min. The ethidium fluorescence (excitation at 490 nm, emission at 610 nm) was examined by fluorescence microscopy.

### 2.7. Lipid Peroxidation Detection

The frozen heart tissues were homogenized in 250 *μ*L of ice-cold 20 mM Tris-HCl (pH 7.4). After centrifugation, 200 *μ*L of supernatant was analyzed for lipid peroxidation products malondialdehyde (MDA)/4-hydroxyalkenals (4-HNE) using a lipid peroxidation assay kit (Calbiochem, Darmstadt, Germany) according to the instructions.

### 2.8. ATP Content Assay

ATP concentration was analyzed using an ATP assay kit (Beyotime, China) according to the manufacturer's instructions. In brief, the heart tissue (20 mg) was homogenized in 150 *μ*L of lysis buffer then centrifuged at 12,000*g* for 10 min at 4°C to collect the supernatant. 100 *μ*L ATP detection working solution and 50 *μ*L samples of the collect supernatant were mixed in the well of 96-well plate, and the luminescence was measured immediately at 562 nm using a microplate reader (ELx800; BioTek Instruments, Winooski, VT, USA). ATP concentration was calculated using a standard curve with known concentrations (0, 10, 100, 200, and 500 *μ*mol/L) of an ATP standard. The relative ATP level was normalized to the total protein according to the following formula: ATP level = ATP concentration/protein concentration.

### 2.9. Real-Time Polymerase Chain Reaction (PCR) for Gene Expression

Total RNA was extracted from tissues using TRIzol (Invitrogen, Carlsbad, CA, USA) and reverse transcribed into cDNA using the PrimeScript RT reagent kit (Takara Biotechnology, Dalian, China) according to the manufacturer's instructions. The mRNA levels of target genes were quantified using SYBR Green Master Mix (Takara Biotechnology) with the ABI PRISM 7500 Sequence Detection System (Applied Biosystems, Branchburg, New Jersey, USA). Each reaction was performed in duplicate, and changes in relative gene expression normalized to GAPDH levels were determined using the relative threshold cycle method. The primer sequences are shown in Supplementary Table 1.

### 2.10. Enzyme-Linked Immunosorbent Assay (ELISA)

Mouse serum was collected from mice at the time of sacrifice in glass tubes and stored at −80°C until use. TNF-*α*, IL-1*β*, and IL-6 were measured using ProcartaPlex® Multiplex Immunoassays (eBioscience, USA) according to manufacturer's instructions.

### 2.11. Mitochondrial Imaging

H9C2 cells were grown in a six-well plate containing 25 mm glass coverslips and treated with 10 *μ*g/mL LPS-EB for 24 h, 20 ng/mL TNF-*α*, 20 ng/mL IL-1*β*, 40 ng/mL IL-6 for 12 h, and 125 *μ*M H_2_O_2_ for 1 h, respectively. H9C2 treatment for the following parts was the same as above. For mitochondrial staining, cells were incubated in 200 nM MitoTracker® Red FM (dissolved in DMSO) (Invitrogen, USA) and preheated in culture medium at 37°C for 30 min. The cells were washed and fixed with 4% paraformaldehyde for 10 min and then washed again and observed under a Nikon Eclipse 80i fluorescence microscopy. An increase in the fragmentation pattern and in mitochondrial number, together with a decrease in mean mitochondrial volume, was considered as criteria for mitochondrial fission. Within cells, two to three regions of interest (ROI) of equal area were defined, mitochondrial counts and volume were measured for each ROI. Each experiment was performed four times, and 16–25 cells/condition were quantified.

### 2.12. Transmission Electron Microscopy

Transmission electron microscopy (TEM) for morphological analysis was performed at Electron Microscopy Core Laboratory, Shanghai Medical College, Fudan University, according to standard operating procedures. For morphological TEM, heart tissue or cell was fixed in 2.5% glutaraldehyde in phosphate buffer overnight at 4°C. After sample preparation, 90–100 nm thick sections were mounted onto a 200 mesh copper grid and imaged with an FEI Tecnai G2 Spirit transmission electron microscope.

### 2.13. Western Blot Analysis

The total lysates from heart tissue were immunoblotted and probed with primary antibodies against DRP1 (BD Biosciences, USA), P-DRP1 (Ser616)/P-DRP1 (Ser637) (Cell Signaling Technology, USA), MFN1/2 (Santa Cruz Biotechnology, USA), OPA1 (BD Biosciences, USA), and internal reference using an adaptation of a previously described protocol [19].

### 2.14. OMA1 Knockdown by shRNA

Four small interfering RNA sequences targeting rat OMA1 without sequences homologous with those of other genes and one scrambled control were designed and inserted into pLOK.1 plasmid (Supplementary Table 2). To package lentiviral particles, 293A cells at 60–70% confluency in six-well plates were transfected for 4 h with 1.0 *μ*g pLKO.1-shOMA1 (functioning vector), 0.75 *μ*g pSPAX2 (packaging vector), and 0.25 *μ*g pMD2.G (packaging vector) using PolyJet (SignaGen, USA) in a serum-free medium. After 4 h, the transfection solution was replaced with a fresh medium. 48 h after medium change, a culture medium containing lentivirus was collected and cell debris was removed by centrifugation. Then, H9C2 cells at 60–70% confluency were infected with above collected lentivirus and selected with puromycin (10 *μ*g/mL). Depletion was confirmed at protein levels, and two-pooled clones were selected and propagated.

### 2.15. Transfection of YME1L Plasmid

Amplified PCR fragments of YME1L from human cDNA bank were purified and inserted into the expression vector pcDNA3.1 according to the manufacturer's protocols (Gibson Assembly Mix, New England Biolabs). The constructed plasmid was propagated and sequenced to confirm the accuracy then transfected to H9C2 and detected the expression efficiency of YME1L by Western blot.

### 2.16. Apoptosis Assay by Flow Cytometry

The amount of apoptosis was determined using the annexin V-EGFP/PI Apoptosis Detection Kit (Vazyme, Nanjing, China) according to the manufacturer's instructions. Briefly, H9C2 cell was treated with 20 ng/mL TNF-*α* for 12 h or 125 *μ*M H_2_O_2_ for 1 h and then stained with EGFP-conjugated annexin V and PI for 10 min at room temperature. The percentage of positive cells collected from over 10,000 events was analyzed by a FACSCalibur system (BD Biosciences, San Jose, CA, USA).

### 2.17. Statistics

The data are presented as the means ± SD unless indicated otherwise. The differences were evaluated using unpaired Student's *t*-tests between two groups when the data were normally distributed and group variances were equal. The Mann–Whitney rank sum test was used when the data were not normally distributed or if group variances were unequal. A one-way analysis of variance (ANOVA) was used for multiple comparisons followed by a post hoc Tukey's procedure for multiple range tests when the data were normally distributed and the group variances were equal. The Kruskal-Wallis test, followed by Dunn's test, was used when the group data were not normally distributed or if the group variances were unequal. GraphPad Prism version 6.0 software was used for statistical analyses, and the statistical significance was set at *p* < 0.05.

## 3. Results

### 3.1. TLR4 Activation Promoted the Progression of Autoimmune Myocarditis to Dilated Cardiomyopathy

**Figure 1:**
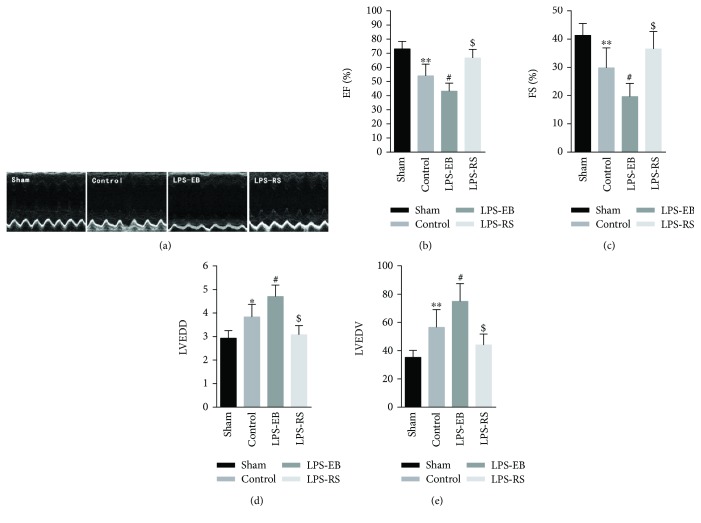
TLR4 activation promoted the progression of autoimmune myocarditis to dilated cardiomyopathy. (a) Representative illustrations of cardiac echo in each group at the end of experiment. (b-c) Ejection fraction (EF, %), fractional shortening (FS, %), LV end-diastolic dimension (LVEDD, mm), and LV end-diastolic volume (LVEDV, *μ*L) were measured after TLR4 intervening in EAM mice (*n* = 6–8). ^∗^ *p* < 0.05, ^∗∗^ *p* < 0.01 versus sham; ^#^ *p* < 0.05 versus control; ^$^ *p* < 0.05 versus control.

Myocardial inflammatory response in EAM mice reaches the peak at the third week, then turning into chronic inflammation phase; continuous inflammation response promotes cardiomyocyte apoptosis, myocardial fibrosis, and declined heart function, eventually resulting in DCM [1, 2]. We previously showed that the expression of myocardium TLR4 was increased in EAM mice, suggesting that TLR4 may be involved in the chronic inflammatory process of EAM [19]. In this study, we found that the cardiac function (EF, FS) was lower in the LPS-EB group than the control group (EF: 43 ± 5.7% versus 54 ± 8.2%, *p* < 0.05; FS: 20 ± 4.7% versus 30 ± 7.0%); meanwhile, the LVEDD and LVEDV were significantly increased (LVEDD: 4.7 ± 0.49 mm versus 3.8 ± 0.53 mm, *p* < 0.05; LVEDV: 74.9 ± 12.5 *μ*L versus 56.5 ± 8.7 *μ*L, *p* < 0.05), indicating a worse ventricular dilatation (Figures 1(a)–1(c)). However, after TLR4 activation was suppressed in the LPS-RS group, the heart function declined and ventricular dilatation was alleviated compared with the control group (EF: 67 ± 6.1% versus 54 ± 8.2%, *p* < 0.05; FS: 37 ± 6.2% versus 30 ± 7.0%, *p* < 0.05; LVEDD: 3.1 ± 0.39 mm versus 3.8 ± 0.53 mm, *p* < 0.05; LVEDV: 44.1 ± 7.6 *μ*L versus 56.5 ± 8.7 *μ*L, *p* < 0.05) (Figures 1(a), 1(d), and 1(e)).

**Figure 2:**
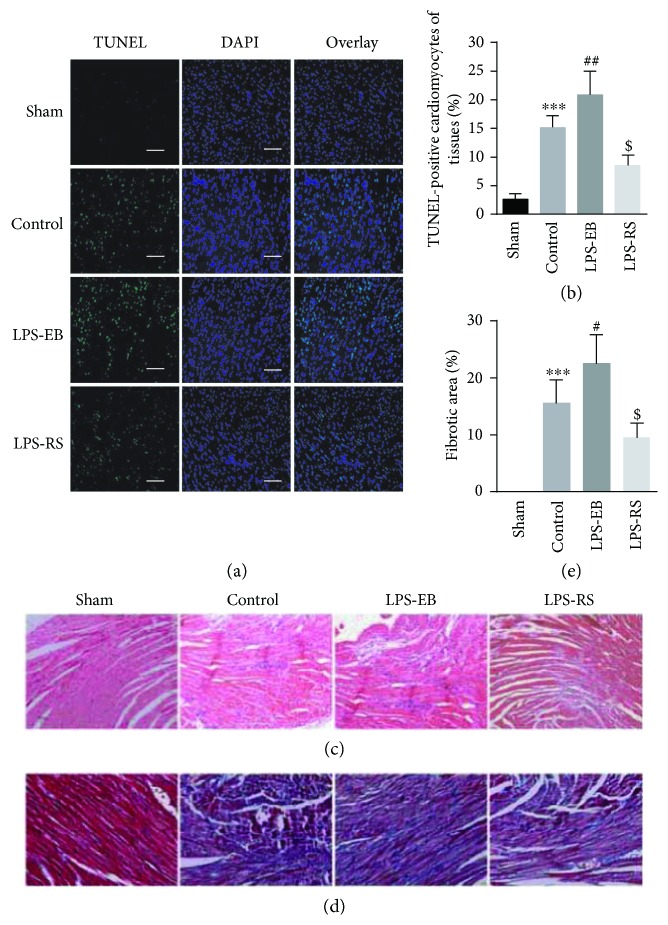
TLR4 activation increased the cardiomyocyte apoptosis and myocardial fibrosis in DCM mice. (a) Apoptotic cell nuclei of cardiac sections in each group were evaluated by terminal deoxynucleotidyl transferase dUTP nick-end labelling (TUNEL) staining (green) and total nuclei by 4′,6-diamidino-2-phenylindole (DAPI) staining (blue). Bar, 50 *μ*m. (b) Quantitative analysis of percentage of cardiomyocytes undergoing apoptosis in vivo (*n* = 6). (c) Representative illustrations of inflammatory cell infiltration in cardiac sections taken by H&E staining (×20). (d) Masson staining of left ventricular tissue slices depicting fibrosis (blue area) (×20). (e) Quantitative analysis showing percentage area of fibrosis (*n* = 5 − 6). ^∗∗∗^ *p* < 0.001 versus sham; ^#^ *p* < 0.05, ^##^ *p* < 0.01 versus control; ^$^ *p* < 0.05 versus control.

As showed in Figures 2(a) and 2(b), cardiomyocyte apoptosis was obviously increased in the control group compared with the sham group (15.3 ± 2.2% versus 2.5 ± 1.0%, *p* < 0.001), and TLR4 activation induced a higher rate of apoptosis in the LPS-EB group compared with the control group (21.0 ± 4.3% versus 15.3 ± 2.2%, *p* < 0.01), but TLR4 inhibition resulted in a decline in the LPR-RS group (8.5 ± 1.9% versus 15.3 ± 2.2%, *p* < 0.01). We further detected the expression of Bcl-2/Bax by RT-PCR and found that the changes were consistent with above results (*p* < 0.05) (Supplementary Figure 1A).

By staining the myocardium with HE, we showed that the inflammatory cells were most serious in the LPS-EB group, which can be largely suppressed in the LPS-RS group, suggesting that TLR4 activation-induced myocardial lesion may be due to regulation of inflammatory cell infiltration (Figure 2(c), Supplementary Figure 1B). An obvious fibrosis is often accompanied by a serious ventricular dilatation [21]. Then, we detected the myocardial fibrosis by Masson staining, showing that the collagen fibers in the LPS-EB group were more than those in the control group (22.3 ± 5.2% versus 15.5 ± 4.1%, *p* < 0.05), while decreased in the LPS-RS group (9.5 ± 2.6% versus 15.5 ± 4.1%, *p* < 0.05) (Figures 2(d) and 2(e)). Lastly, we observed that the heart/body weight ratio (H/B) was increased in control group compared with the sham group (5.2 ± 0.75 versus 4.1 ± 0.39, *p* < 0.05), and TLR4 activation showed a higher ratio in the LPS-EB group (6.2 ± 0.90 versus 5.2 ± 0.75, *p* < 0.05), but TLR4 inhibition resulted in a decrease in the LPS-RS group (Supplementary Figure 1C). These results indicate that TLR4 activation promoted the transition of EAM to DCM by regulating cardiomyocyte apoptosis and myocardial fibrosis.

### 3.2. TLR4 Activation Increased the Oxidative Stress and Proinflammatory Cytokine Level in DCM Mice

**Figure 3:**
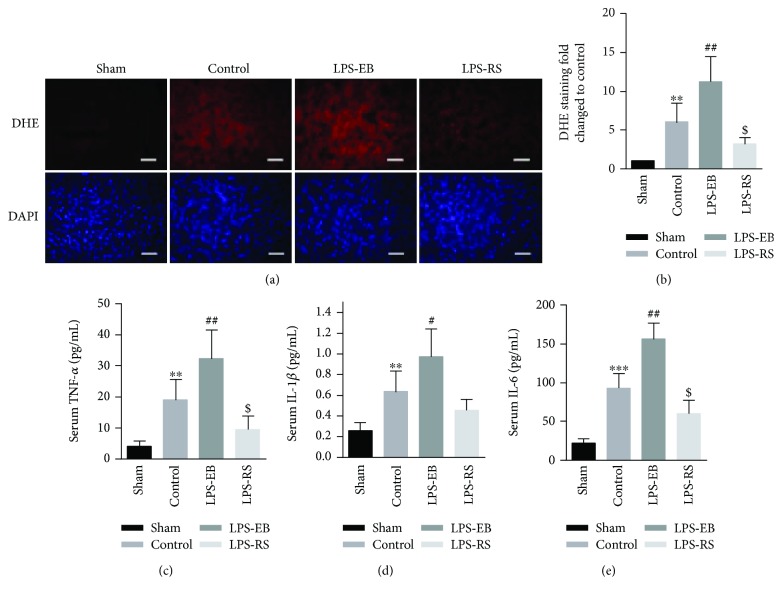
TLR4 activation increased the oxidative stress and proinflammatory cytokine level in DCM mice. (a) ROS production in the cardiac sections was evaluated with dihydroethidium (DHE) staining. Bar, 20 *μ*m. (b) Quantitative analysis of ROS as normalized to the sham group (*n* = 5 − 6). (c–e) Serum levels of TNF-*α* (c), IL-1*β* (d), and IL-6 (e) were measured by Multiplex Immunoassays (*n* = 6). ^∗∗^ *p* < 0.01, ^∗∗∗^ *p* < 0.001 versus sham; ^#^ *p* < 0.05, ^##^ *p* < 0.01 versus control; ^$^ *p* < 0.05 versus control.

TLR4 activation can increase the infiltration of inflammatory cells in the myocardium, upregulating the expression of inflammatory factors and promoting the production of ROS, which corporately results in cardiomyocyte apoptosis and myocardial fibrosis, and thereby promoting DCM progression [1, 4, 22]. As shown in Figures 3(a) and 3(b), ROS stress in the control group significantly increased compared with the sham group, and TLR4 activation further intensified ROS production in myocardial tissue in the LPS-EB group; however, TLR4 inhibition suppressed the produce in the LPS-RS group. Consistent with the above result, the ROS-producing activity of cardiac homogenates in these groups, as evaluated with lipid peroxides (malondialdehyde and 4-hydroxyalkenals), showed the similar changes (Supplementary Figure 1D).

Next, we detected the levels of the above cytokines in the serum of each group. Compared with the sham group, these cytokines were significantly increased (*p* < 0.01). The level was further elevated after TLR4 activation in the LPS-EB group (*p* < 0.05) and downregulated after TLR4 inhibition in the LPS-RS group (Figures 3(c)–3(e)). The transcription level of the above cytokines in the myocardium was detected by RT-PCR, which showed a consistent expression level as in serum (*p* < 0.05) (Supplementary Figure 1E). These results suggest that TLR4 activation may promote the myocardium ROS stress and upregulate the expression of inflammatory cytokines such as TNF-*α*, IL-1, and IL-6, which involved in the progression of EAM to DCM in mice.

### 3.3. Mitochondrial Dynamic Imbalance Was Increased in DCM Mice after TLR4 Activation

**Figure 4:**
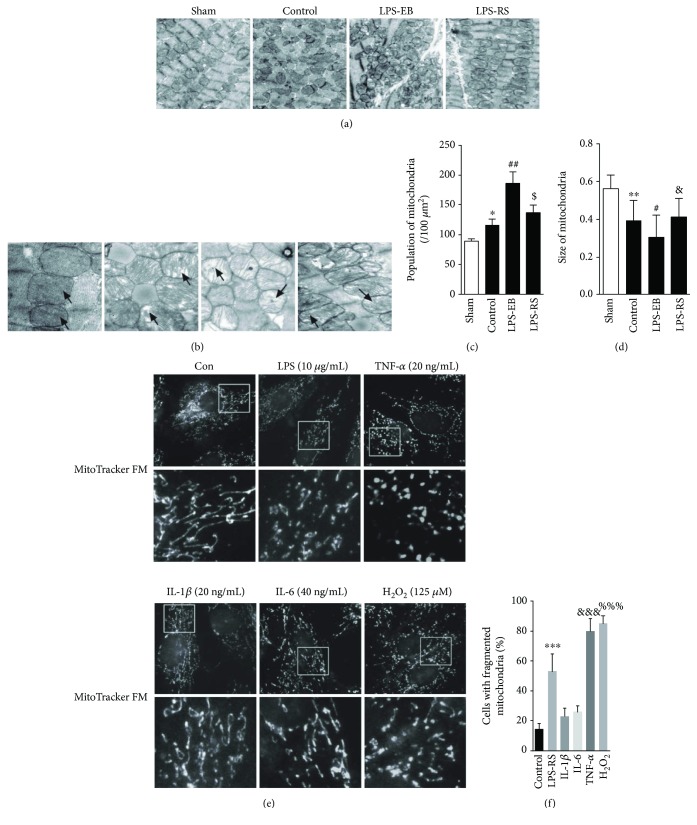
Mitochondrial dynamic imbalance was increased in DCM mice after TLR4 activation. (a) Representative illustrations of transmission electron microscopy (TEM) showing the fragmentation of mitochondria in each group (×6000). (b) Representative illustrations of transmission electron microscopy (TEM) showing the ultrastructural changes of mitochondria in each group (×11,000). (c-d) Graphs summarize the amount of mitochondria per area and average mitochondrial size of mitochondria (*n* = 4). (e) Representative images of mitochondrial morphology in H9C2. (f) Quantification of four independent experiments for fragmented mitochondria (*n* = 4). Bar = 2 *μ*m. ^∗^ *p* < 0.05, ^∗∗^ *p* < 0.01 versus sham; ^#^ *p* < 0.05, ^##^ *p* < 0.01 versus control; ^$^ *p* < 0.05 versus control; ^&^ *p* > 0.05 versus control (c-d); ^∗∗∗^ *p* < 0.001, ^&&&^ *p* < 0.001, ^%%%^ *p* < 0.001 versus control (f).

We have showed that TLR4 activation can promote the progress of DCM in EAM mice; next, we observed if mitochondrial dynamics was involved in it. As shown in Figures 4(a) and 4(b) by transmission electron microscopy (TEM), the mitochondria were closely spaced each other in a line, which was parallel to the sarcomere orientation in the sham group, and the mitochondrion was elliptical with dark and dense mitochondrial crista, no visible vacuole formed. However, in the control group, the mitochondria became arranged in disorder, small, and fragmented, furthermore swollen and vacuolated partly, and also with damaged crista in ultrastructure. Compared with the control group, above mitochondrial aberration in structure showed more serious in the LPS-EB group but was mitigated in the LPS-RS group. The absolute number of mitochondria per area was obviously increased, and the individual mitochondrial cross-sectional area was significantly decreased in control and LPS-EB groups compared with the sham group, but not decreased in the LPS-RS group (Figures 4(c) and 4(d)). For the mitochondrial function, we evaluated ATP concentration of heart tissue with ATP kit and found the ATP level reduced in the control group and worsened significantly in LPS-EB groups (Supplementary Figure 2). These results suggest that mitochondrial dynamic imbalance and ultrastructural damage may be involved in the pathophysiological process of DCM. Next, we investigated the effect of TLR4 activation on mitochondrial dynamics in H9C2 cardiomyocytes. We found that the mitochondrial fission was increased in H9C2 treated with LPS-EB (Figures 4(c) and 4(d)). As TLR4 activation was found to lead to myocardial injury mainly by upregulating proinflammatory cytokines and inducing ROS stress, so we observed the mitochondrial dynamics of H9C2 cardiomyocytes treated with TNF-*α*, IL-1*β*, IL-6, and H_2_O_2_. As showed in Figures 4(c) and 4(d), TNF-*α* and H_2_O_2_ treatment increased the mitochondrial fission, but IL-1*β* and IL-6 had no obvious effect by MitoTracker Red staining. Beside, we also showed that TNF-*α* may promote the mitochondrial fission by directly inducing the mitochondrial oxidative stress (Supplementary Figure 3). In vivo and in vitro experiments suggest that mitochondrial dynamic imbalance of cardiomyocytes in DCM may not be only directly mediated by LPS-EB stimulation but also by TLR4 activation-induced inflammatory response, such as upregulated inflammatory factors (TNF-*α*) and ROS stress.

### 3.4. OPA1 Dysfunction Involved in Mitochondrial Dynamic Imbalance and Damage in DCM Mice after TLR4 Activation

**Figure 5:**
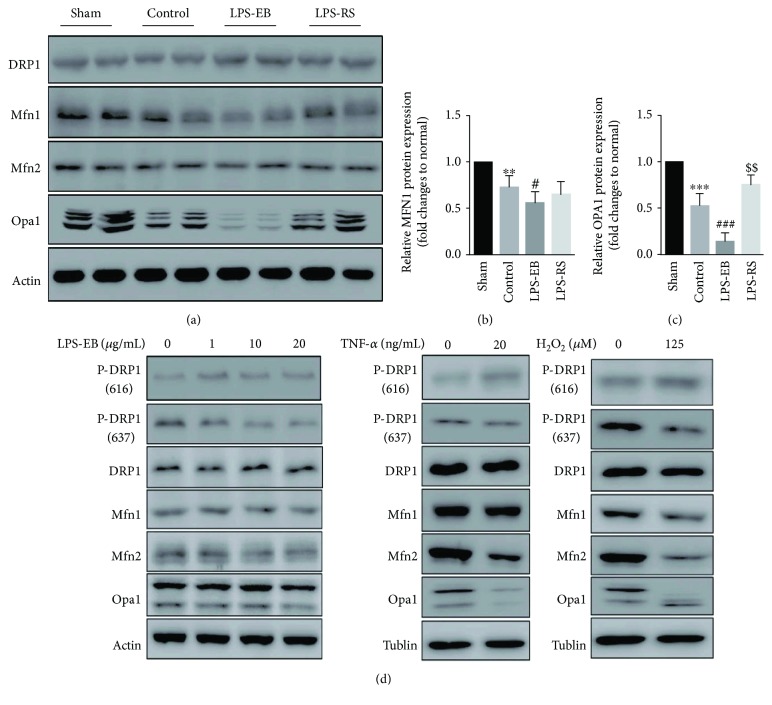
OPA1 dysfunction involved in mitochondrial dynamic imbalance and damage in DCM mice after TLR4 activation. Representative Western blot (a) and quantitative analysis (b-c) of mitochondrial dynamic-related molecule expression in myocardium of each group (*n* = 5). (d) Representative Western blot of mitochondrial dynamic-related molecule expression in H9C2 treated with LPS-EB, TNF-*α*, and H_2_O_2_ (*n* = 4). ^∗∗^ *p* < 0.01, ^∗∗∗^ *p* < 0.001 versus sham; ^#^ *p* < 0.05, ^###^ *p* < 0.001 versus control; ^$$^ *p* < 0.01 versus control.

To investigate the molecular mechanism about the mitochondrial dynamic imbalance, we detected the levels of related molecules including DRP1, MFN1, MFN2, and OPA1. We showed that the phosphorylation of DRP1 and the expression of MFN2 appeared not obviously changed, and the expression of MFN1 and OPA1 appeared obviously changed, but OPA1 more significantly; furthermore, above molecule change was regulated by TLR4 activation in LPS-EB and LPS-RS groups, as revealed that TLR4 activation obviously promoted OPA1 downregulation and TLR4 inhibition increased the level of OPA1 compared with the control group (Figures 5(a)–5(c)). Next, we further detected the expression of above molecules in H9C2 cardiomyocytes. In LPS-EB-stimulated H9C2, the level of phosphorylation in DRP1 637 was decreased, and the level of phosphorylation in DRP1 616 was increased. But the expression of MFN1, MFN2, and OPA1 did not change obviously, indicating that LPS-EB stimulation-mediated mitochondrial fission was due to DRP1 activation in H9C2. In TNF-*α*-stimulated H9C2, the level of phosphorylation in DRP1 616 and 637 did not change significantly, while the level of OPA1 and MFN2 was decreased. However, in H_2_O_2_-treated H9C2, all above molecules were affected significantly (Figure 5(d)). The downregulated OPA1 level in TNF-*α* and H_2_O_2_ treatment was consistent with the obvious damage in the ultrastructure of mitochondria (Supplementary Figure 4A), as OPA1 was reported to influence not only the mitochondrial dynamics but also mitochondrial crista stabilization [23]. These results suggest that TLR4 activation mainly promoted the OPA1 downregulation, causing mitochondrial dynamic imbalance and damage by regulating inflammatory response, such as upregulated inflammatory factors (TNF-*α*) and ROS stress in the progression of DCM.

### 3.5. OMA1/YME1L Abnormal Degradation Mediated OPA1 Dysfunction in DCM Mice after TLR4 Activation

**Figure 6:**
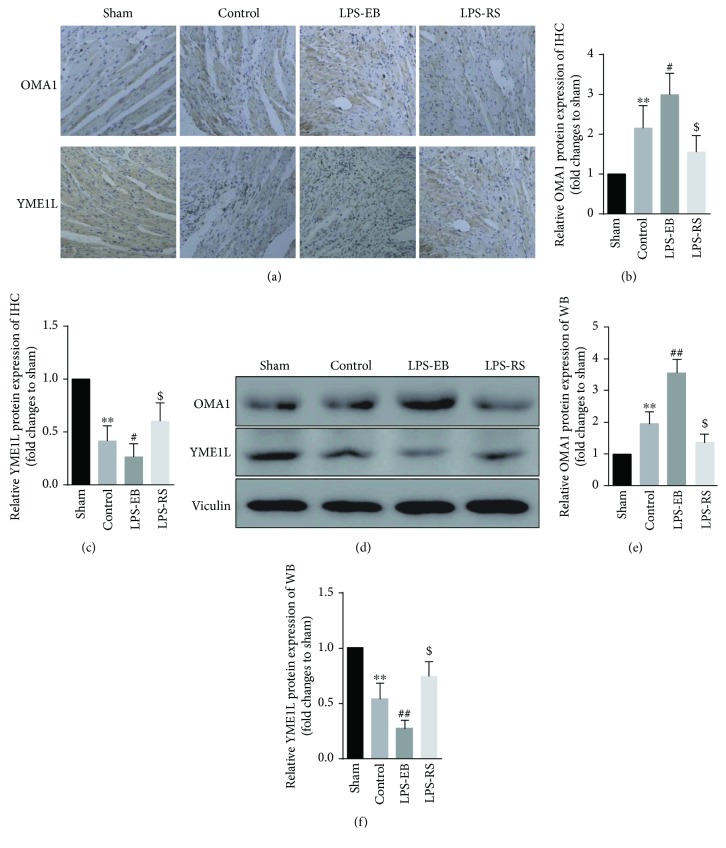
OMA1/YME1L abnormal degradation mediated OPA1 dysfunction in DCM mice after TLR4 activation. Immunohistochemistry (×20) (a) and quantitative analysis (b-c) of expression of OMA1 and YME1L of myocardium in each group (*n* = 5 − 6). Representative Western blot (d) and quantitative analysis (e-f) of expression of OMA1 and YME1L of myocardium in each group (*n* = 5 − 6). ^∗∗^ *p* < 0.01 versus sham; ^#^ *p* < 0.05, ^##^ *p* < 0.01 versus control; ^$^ *p* < 0.05 versus control.

Our results showed that TLR4 activation promoted the mitochondrial dynamic imbalance and ultrastructure damage in DCM mice by mainly inducing OPA1 dysfunction. OPA1 was found to be cleaved by mitochondrial proteases YME1L and OMA1, whose abnormal activation would result in OPA1 dysfunction and degradation, playing an important role in the pathophysiology of diseases [24, 25]. Therefore, we next detected the expression of OMA1 and YME1L in the cardiac tissue of each group. By immunohistochemistry, the expression of OMA1 in the control group was higher than that in the sham group (*p* < 0.01), and the expression of OMA1 was increased obviously in the LPS-EB group compared with the control group (*p* < 0.05), while its expression was inhibited in the LPS-RS group (*p* < 0.05); compared with the Sham group, the expression of YME1L in the control group was significantly decreased (*p* < 0.01), and the level of YME1L was further decreased in the LPS-EB group compared with the control group (*p* < 0.05), while the downregulated expression of YME1L was inhibited in the LPS-RS group (*p* < 0.05) (Figures 6(a)–6(c)). We observed the consistent changes in measuring OMA1 and YME1L by Western blot (Figures 6(d)–6(f)). These results indicate that the OMA1 activity may be enhanced, but YME1L activity was weakened, which led to dysfunctional OPA1 processing.

**Figure 7:**
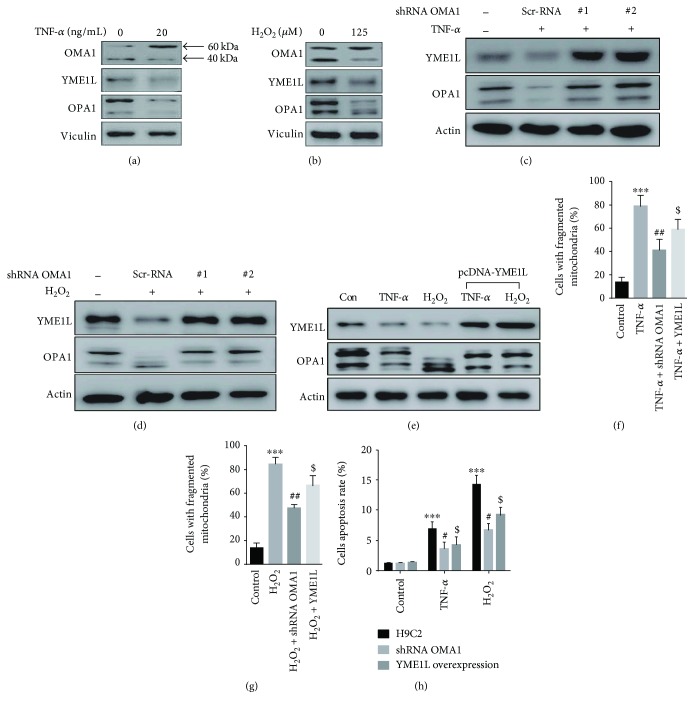
Intervening OMA1 and YME1L suppressed mitochondrial fission and apoptosis in H9C2. (a-b) Representative immunoblots of OMA1, YME1L, and OPA1 in H9C2 treated with TNF-*α* and H_2_O_2_. (c-d) The effect of OMA1 knockdown on the level of YMEL1 and OPA1 in H9C2 treated with TNF-α and H_2_O_2_. (e) The effect of YME1L overexpression on the level of YMEL1 and OPA1 in H9C2 treated with TNF-*α* and H_2_O_2_. (f-g) Quantification of four independent experiments for mitochondrial morphology in H9C2 treated with TNF-*α* and H_2_O_2_. (h) The effect of OMA1 knockdown or YME1L overexpression on apoptosis in H9C2 treated with TNF-*α* and H_2_O_2_ with flow cytometry (*n* = 4 − 5). ^∗∗∗^ *p* < 0.001 versus control; ^#^ *p* < 0.05, ^##^ *p* < 0.01 versus H9C2 treated with TNF-*α* or H_2_O_2_ group; ^$^ *p* < 0.05 versus H9C2 treated with TNF-*α* or H_2_O_2_ group.

In in vitro experiment of H9C2 cardiomyocytes, we found that TNF-*α* and H_2_O_2_ treatment promoted OPA1 downregulation, accompanied with an increase of band at about 60 kDa and a significant reduction of band at about 40 kDa in OMA1, which signified the activation of OMA1, and the downregulation of YME1L, which indicated the dysfunction of YME1L (Figures 7(a) and 7(b)). In addition, in H9C2 with OMA1 knockdown (Supplementary Figure 4B), we showed that the OPA1 processing was obviously suppressed after treatment with TNF-*α* and H_2_O_2_ (Figures 7(c) and 7(d)); also by overexpressing YME1L in H9C2 (Supplementary Figure 4C), we observed that the OPA1 processing was obviously suppressed after treatment by TNF-*α* and H_2_O_2_ (Figure 7(e)). Next, we investigated the mitochondrial dynamics in TNF-*α* or H_2_O_2_-treated H9C2 by OMA1 knocking down or YME1L overexpression. Both the interventions presented decreased mitochondrial fission in H9C2 mediated by TNF-*α* or H_2_O_2_ (Figures 7(f) and 7(g)). Furthermore, by cell TEM, both OMA1 and YME1L interventions significantly alleviated the mitochondrial ultrastructure damage (Supplementary Figure 4A). In normal condition, OMA1 knocking down or YME1L overexpression did not influence the apoptosis in H9C2; however, both two interventions obviously decreased the apoptosis rate in H9C2 mediated by TNF-*α* or H_2_O_2_ (Figure 7(h)). These results suggest that TNF-*α* and H_2_O_2_ contributed to the mitochondrial damage and cell apoptosis by regulating OMA1 and YME1L abnormal activation and OPA1 dysfunction, which may be associated with the TLR4 activation-mediated DCM progression in EAM mice.

## 4. Discussion

In this study, we report for the first time that TLR4 activation aggravated the mitochondrial dynamic imbalance, promoting the progression from EAM to DCM. Our results demonstrated that TLR4 activation mainly led to OPA1 dysfunction, which accompanied by increased cardiomyocyte apoptosis, myocardial fibrosis, ventricular dilatation, and declined heart function. Mechanistically, we showed that TLR4 activation elevated TNF-*α* level and ROS stress, which may mediate OMA1/YME1L abnormal degradation and OPA1 dysfunction, jointly led to mitochondrial dynamic imbalance and damage, eventually promoting the progression from EAM to DCM in mice.

Mitochondria appear highly dynamic, constantly undergoing fusion and fission to adapt to the changes of cellular environment, which is crucial for compensating for mitochondrial damage [13]. An increasing number of studies have shown that mitochondrial dynamics can be regulated and involved in the pathophysiology of inflammatory diseases [26]. Mitochondrial dynamic abnormity and damage are closely related with the LPS stimulation-mediated tissue injury and organ failure including lung, heart, liver, and so on [27]. Mitochondrial damage could be obviously alleviated by antioxidative treatment, such as thioredoxin, CO, and heme oxygenase [28–30], indicating that ROS stress was the main cause of mitochondrial dynamic imbalance. Dysfunctional mitochondrial dynamics restricted the mitophagy progress for segregation of unrecoverable damaged mitochondria, characteristic with destabilized protein accumulation and mtDNA copy number decrease [31]. Consistent with the study conducted by Chen et al. who showed increased mitochondrial fission in the explanted failing human heart [17], we observed obviously disorganized and fragmented mitochondria, moreover, mitochondrial cristae damage in the cardiomyocytes of DCM mice. The damaged mitochondria produce more ROS and less ATP and present a lower threshold for cytochrome c release and mitochondrial permeability transition pore (MPTP) opening in cell apoptosis; furthermore, these changes also induce the release of mitochondrial components (mtHSP60, mtDNA) into cytosol identified by receptors for damage-associated molecular patterns (DAMP) and activate inflammation response in a vicious cycle [31, 32]. In another study, Ikeda et al. reported that mitochondrial fission imbalance and damage can be alleviated by adequate drug treatment and left ventricular assist device, accompanied with significantly improved cardiac function in dilated cardiomyopathy patients [33], so the mitochondrial dynamic balance closely relates with cardiac pathophysiology.

LPS stimulation has been showed to promote cell apoptosis, related with upregulated inflammatory cytokine expression, ROS level, and mitochondrial dysfunction [27]. LPS stimulation-mediated cardiac suppression was closely associated with leukocyte activation, instead of the direct effect of TLR4 activation on cardiomyocytes [27, 34]. So the OPA1 dysfunction in DCM may be associated with the indirect effect of TLR4 activation-induced inflammation response. Previous studies have shown that TNF-*α*, IL-1*β*, IL-6, and ROS stress could influence mitochondrial dynamics [35, 36]. However, we found that only TNF-*α* and ROS stress can promote mitochondrial fission and mitochondria ultrastructure injury in H9C2 cells, whereas IL-1*β* and IL-6 have no significant effect; the mechanism of which may mainly relate with OPA1 and MFN2 dysfunction. In adipocyte, TNF-*α* can promote mitochondrial fragmentation dependent on OPA1 downregulation; nevertheless, IL-6 and IL-1*β* showed no effect on mitochondrial dynamics [36]. In cultured C2C12 myoblasts, IL-6 had increased FIS1 protein expression, increased oxidative stress, and reduced PGC-1*α* gene expression, which suppressed MFN2 expression and promoted mitochondrial fission [35]. So the effect of inflammatory factors on mitochondrial dynamic possesses obvious cell specificity. Besides, we found that the change of OPA in myocardium of DCM mice was not consistent with the finds of LPS stimulation in vitro. The discrepancy between them may result from the difference of selected H9C2 cell and also the stronger role of TLR4 activation-mediated inflammation response [34, 36, 37]. Therefore, in our DCM model, TLR4 activation may promote the mitochondrial fission and damage by upregulating the inflammatory factors (mainly TNF-*α*) and ROS stress; the molecular mechanism of which entails further investigation.

A balance of long and short forms of OPA1 is crucially important for its functions, which are regulated by complex patterns of alternative splicing and proteolysis [38]. Mitochondrial proteases YME1L and OMA1 have been found to relate with constitutive and stress-induced OPA1 processing [39]. Loss of OMA1 blocks OPA1 processing but does not grossly influence mitochondrial morphology, while depletion of YME1L impairs constitutive processing of OPA1 and leads to mitochondrial fission, disorganizes cristae morphogenesis, and renders cells impressionable to apoptosis stimuli [39, 40]. Moreover, the reciprocal regulation and degradation of YME1L and OMA1 existed, namely in mitochondrial depolarization and metabolic stress condition, OMA1 was activated, and YME1L was degraded with OMA1 dependency; however, in normal condition, the activity of OMA1 was suppressed by a way which may depend on YME1L [23, 40]. Studies have showed that OMA1 activation made all long form of OPA1 cleaved, thereby disrupting the balance of long and short forms, which involved in ischemia-induced tissue injury of kidney and brain in animal models [24, 25]. Wai et al. also showed that imbalanced OPA1 processing and mitochondrial fragmentation caused dilated cardiomyopathy and heart failure in cardiomyocyte-specific YME1L knockout mice [41]. In this study, we found that OMA1 level was increased in the LPS-EB group compared with the control group. However, in H9C2, the level of OMA1 in about 60 kDa was increased, but the level of OMA1 in about 40 kDa was decreased. Head et al. reported that a 60 kDa OMA1 precursor was firstly translated in the cytosol and cleaved upon mitochondrial import to a 40 kDa mature form by an unidentified protease yet [42]. Furthermore, mature OMA1 was demonstrated to be degraded in an autocatalytic way when facing mitochondrial stress [43]. So our upregulated level of OMA1 may be as a result of the accumulated precursor because of the mitochondrial dysfunction-induced import decline [44]. The YME1L level was obviously decreased in the LPS-EB group, further confirming the activation of OMA1 and autocatalytic degradation [45]. In addition, we showed the protective effect on mitochondrial dynamics and cristae morphogenesis and the decreased apoptosis rate induced by TNF-*α* or H_2_O_2_ by knockdown of OMA1 or by overexpressing YME1L in H9C2. So the abnormity of YME1L and OMA1 degradation was associated with the DCM progression, which implies a potential therapeutic target in inflammatory cardiomyopathy.

The molecular mechanism of OMA1 activation remains unclear, an in-depth study of the regulation ways and protein structure of OMA1 might thus contribute to exciting therapeutic potential for human diseases related with mitochondrial dysfunction [23, 25, 41]. Facing various stress insults, studies have reported that ischemia, depolarization of the mitochondrial membrane potential, and ROS stress resulted in loss of long OPA1 in an OMA1-dependent manner [40, 45]. These stresses are interconnected and showed to result in mitochondrial dysfunction characteristic with morphology change, cristae ultrastructure damage, oxidative phosphorylation injury, ATP produce decline, and proapoptotic protein release, such as cytochrome c, which initiates the irreversible apoptotic cascade [32]. Intervening above insults has been proved to resist the mitochondrial injury and play a protective role in the pathophysiology process of many diseases [46]. The method of lowering ROS level has been found to possess a protective role on OPA1 dysfunction-induced diseases [47]. Recently, studies have also showed that other compositions in mitochondrial membrane, such as PHB2 and HIGD-1a, modulated the proteolytic activity of OPA1 mediated by proteases, which influenced the mitochondrial fission and disorganization of cristae [48, 49]. Therefore, a further study of the molecular mechanism of OPA1 dysfunction may greatly contribute to the targeted therapy of related diseases such diabetes and neurodegenerative and cardiovascular diseases [23].

There is accumulating evidence that mitochondrial dysfunction is an important contributor to both development of heart diseases and aging process, which are partly due to the reduced mitochondrial quality control (MQC) [50]. The present study acknowledges a limitation that should be stated, we just observed the mitochondrial dynamic change in the pathophysiology process of the transition of autoimmune myocarditis to DCM induced by TLR4 activation, and other aspects of MQC were not included in this study, such as antioxidant system, mitochondrial unfolded protein response, and autophagy (mitophagy), which all are intimately linked to mitochondrial dynamics. So the integral MQC change and the critical molecular mechanism should be focused in future research, and better pharmacological interventions should be presented.

## 5. Conclusion

The present study showed that TLR4 activation was involved in the progression from EAM to DCM. TLR4 activation upregulated the inflammatory factors (mainly TNF-*α*) and ROS stress and resulted in OPA1 dysfunction by inducing OMA1 and YME1L abnormal degradation, which may aggravate the mitochondrial dynamic imbalance and damage, promoting the progression of EAM to DCM.

## Supplementary Materials

Supplementary 1 Figure 1A: the mRNA ratio of Bcl-2/Bax in the heart tissue of each group (*n* = 4). B: Myocarditis severity scores in the heart sections of each group (*n* = 5 − 6). C: The heart/body weight ratio (H/B) in each group (*n* = 6–8). D: Malonaldehyde + 4-hydroxyalkenal (MDA + 4HAE) contents in myocardium of each group were evaluated with the lipid peroxidation detection (*n* = 5 − 6). E: Expression levels of TNF-*α*, IL-1*β*, and IL-6 in myocardium of each group by reverse transcription polymerase chain reaction (RT-PCR) (*n* = 6). ^∗^ *p* < 0.05, ^∗∗^ *p* < 0.01, ^∗∗∗^ *p* < 0.001 versus Sham; ^#^ *p* < 0.05, ^##^ *p* < 0.01 versus control; ^$^ *p* < 0.05 versus control.

Supplementary 2 Figure 2: the ATP levels in the heart tissue of each group (*n* = 5). ^∗^ *p* < 0.05 versus Sham; ^##^ *p* < 0.01 versus control; ^$^ *p* < 0.05 versus control.

Supplementary 3 Figure 3: TNF-*α* induce the mitochondrial oxidative stress in H9C2. A: Representative images of mitochondrial ROS measured by MitoSOX Red fluorescence in H9C2 cells. B: Quantitative analysis of mitochondrial ROS from H9C2 induced by TNF-*α* with or without TEMPO, as normalized to control (*n* = 4). ^&^ *p* > 0.05 versus control, ^∗∗∗^ *p* < 0.001 versus control, ^##^ *p* < 0.01 versus TNF-*α* group.

Supplementary 4 Figure 4A: the ultrastructure change of mitochondria in H9C2 treated with TNF-*α* or H_2_O_2_ by cell transmission electron microscopy (TEM). B-C: Representative immunoblots confirming OMA1 knockdown in OMA1-shRNA-transfected H9C2 and YME1L overexpression in H9C2.

Supplementary 5 Table 1.

Supplementary 6 Table 2: the sequence for shRNA OMA1 is in the following format. The two ends are restriction sites, AgeI on 5′ and EcoRI site on the 3′.
