# Supplementary figures and images for: TLR4 Activation Promotes the Progression of Experimental Autoimmune Myocarditis to Dilated Cardiomyopathy by Inducing Mitochondrial Dynamic Imbalance
Source: Oxid Med Cell Longev. 2018 Jun 26;2018:3181278. doi: 10.1155/2018/3181278 (PMC6038665; doi:10.1155/2018/3181278)

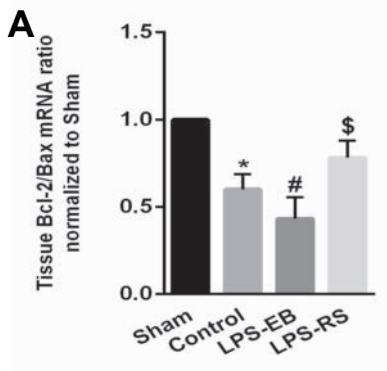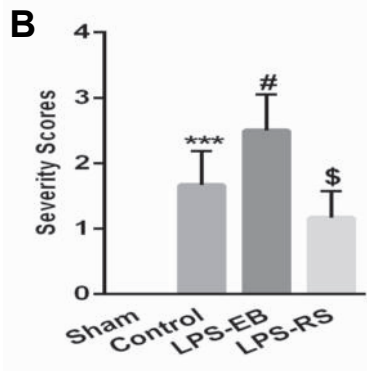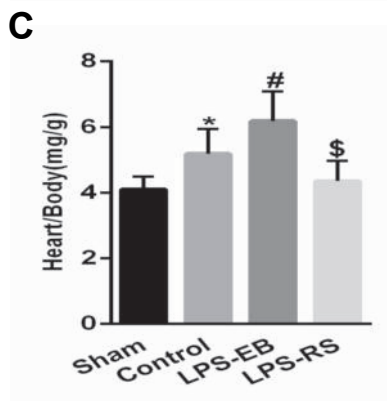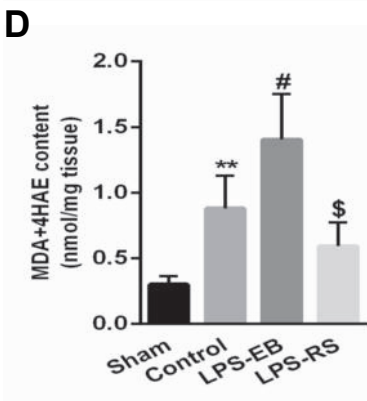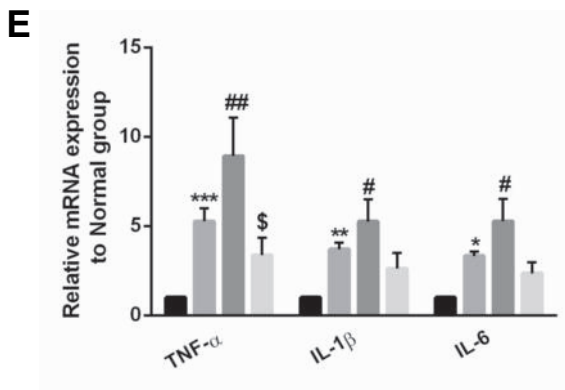

Supplement: Supplementary 1 — Figure 1A: the mRNA ratio of Bcl-2/Bax in the heart tissue of each group (n = 4). B: Myocarditis severity scores in the heart sections of each group (n = 5 − 6). C: The heart/body weight ratio (H/B) in each group (n = 6–8). D: Malonaldehyde + 4-hydroxyalkenal (MDA + 4HAE) contents in myocardium of each group were evaluated with the lipid peroxidation detection (n = 5 − 6). E: Expression levels of TNF-α, IL-1β, and IL-6 in myocardium of each group by reverse transcription polymerase chain reaction (RT-PCR) (n = 6). ∗ p < 0.05, ∗∗ p < 0.01, ∗∗∗ p < 0.001 versus Sham; # p < 0.05, ## p < 0.01 versus control; $ p < 0.05 versus control. [file 3181278.f1.pdf]

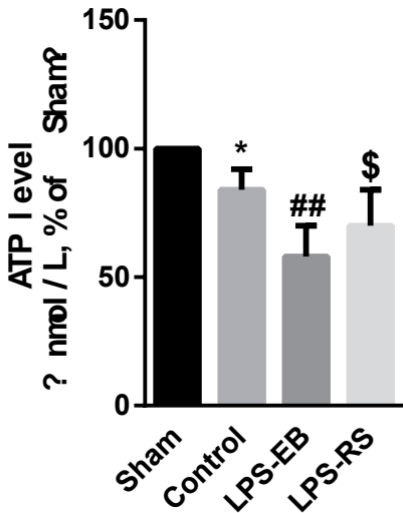

Supplement: Supplementary 2 — Figure 2: the ATP levels in the heart tissue of each group (n = 5). ∗ p < 0.05 versus Sham; ## p < 0.01 versus control; $ p < 0.05 versus control. [file 3181278.f2.pdf]

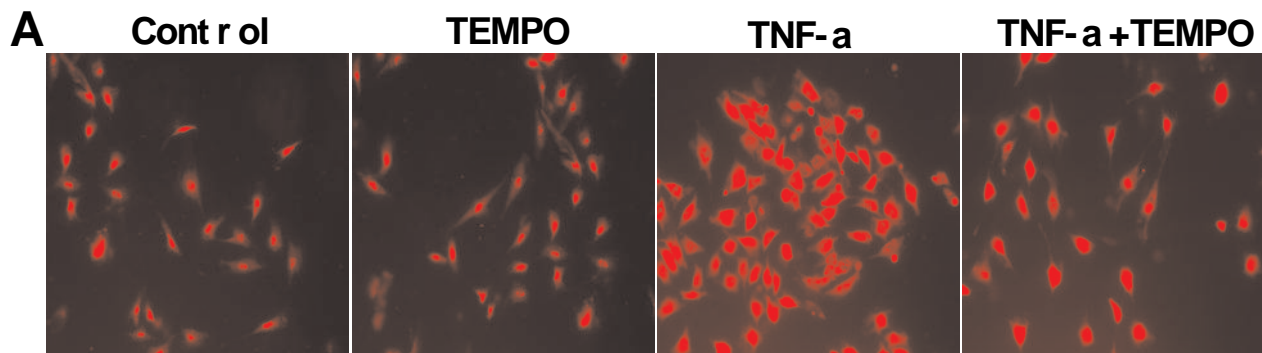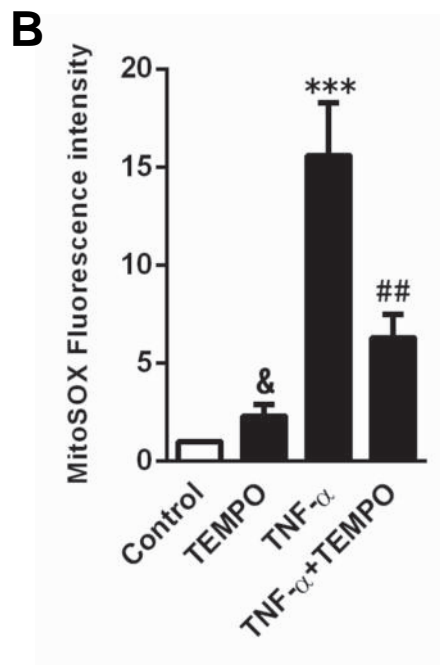

Supplement: Supplementary 3 — Figure 3: TNF-α induce the mitochondrial oxidative stress in H9C2. A: Representative images of mitochondrial ROS measured by MitoSOX Red fluorescence in H9C2 cells. B: Quantitative analysis of mitochondrial ROS from H9C2 induced by TNF-α with or without TEMPO, as normalized to control (n = 4). & p > 0.05 versus control, ∗∗∗ p < 0.001 versus control, ## p < 0.01 versus TNF-α group. [file 3181278.f3.pdf]

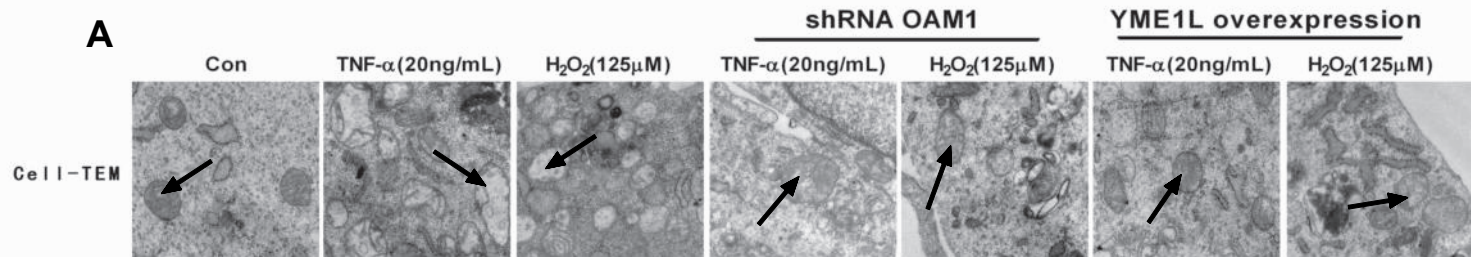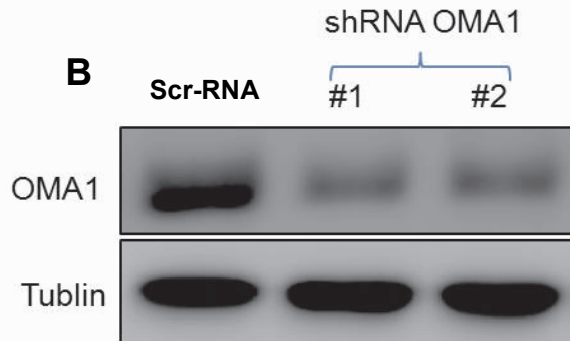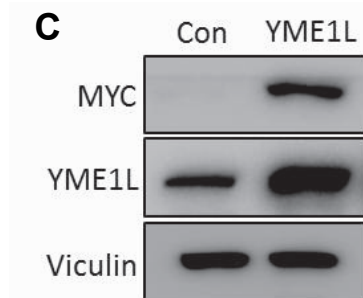

Supplement: Supplementary 4 — Figure 4A: the ultrastructure change of mitochondria in H9C2 treated with TNF-α or H2O2 by cell transmission electron microscopy (TEM). B-C: Representative immunoblots confirming OMA1 knockdown in OMA1-shRNA-transfected H9C2 and YME1L overexpression in H9C2. [file 3181278.f4.pdf]
